# DNA barcoding as a valuable tool for delimiting mollusk species of the genus *Biomphalaria* Preston, 1910 (Gastropoda: Planorbidae)

**DOI:** 10.3389/fcimb.2023.1167787

**Published:** 2023-04-24

**Authors:** Amanda Domingues de Araújo, Omar dos Santos Carvalho, Sandra Grossi Gava, Roberta Lima Caldeira

**Affiliations:** Helminthology and Medical Malacology Research Group, René Rachou Institute, Fiocruz Minas, Belo Horizonte, Minas Gerais, Brazil

**Keywords:** DNA barcode, mollusks, *Biomphalaria*, medical malacology collection, molecular idenlification, cytochrome c oxidase *coi*

## Abstract

**Introduction:**

The genus *Biomphalaria* in Brazil includes 11 species and one subspecies, three of which are intermediate hosts of *Schistosoma mansoni*. Due to the recent evolution of this group, some species are difficult to identify based on morphological characters, making the use of genetic markers necessary for species identification. This study aimed to evaluate the use of partial sequences of the cytochrome c oxidase I (*coi*) gene for the identification of *Biomphalaria* species using phylogenetic reconstruction and species delimitation algorithms. The study tested the use of DNA barcoding technique for species delimitation within the genus.

**Methods:**

DNA barcoding was performed by sequencing a partial region of the *coi* gene from specimens, and the sequences were analyzed using phylogenetic reconstruction and algorithms to delimit Operational Taxonomic Units (OTUs).

**Results:**

The study found that the use of the *coi* gene in the reconstruction of the phylogeny of the genus might be an alternative for understanding the evolution and dispersion of species. However, this marker alone is not enough to solve complex taxonomic problems within the genus. A total of 223 sequences were analyzed, 102 of which could be separated using the barcode gap, enabling the correct identification of seven taxa.

**Discussion:**

The study demonstrated that accurate mollusk identification is necessary for effective schistosomiasis control. The DNA barcoding methodology was found to be promising for accurate mollusk identification, which is crucial for concentrating schistosomiasis control efforts in places where it is needed.

## 1 Introduction

*Schistosoma mansoni* Sambon, 1907 is the etiologic agent of schistosomiasis mansoni, a disease that affects about 130 million people in South America, the Caribbean, Africa, Madagascar, and the Middle East (James et al., 2018; McManus et al., 2018).

Mollusks of the genus *Biomphalaria* (Preston, 1910) belong to the family Planorbidae, which has approximately 300 species, of which about 10% are within the *Biomphalaria* genus (Jarne et al., 2010). There are 26 morphospecies and one subspecies of the latter genus described in Latin America. In Brazil, in particular, there are 11 species and one subspecies, of which three are intermediate hosts of *S. mansoni* under natural conditions – *Biomphalaria glabrata* (Say, 1818), *Biomphalaria tenagophila* (d’Orbigny, 1835) and *Biomphalaria straminea* (Dunker, 1835). 1848) – while another three are considered potential hosts, as they can be infected under laboratory conditions – *Biomphalaria peregrina* (d’Orbigny, 1835), *Biomphalaria amazonica* Paraense, 1966 and *Biomphalaria cousini* Paraense, 1966 (Corrêa, 1971; Paraense and Corrêa, 1973; Teodoro et al., 2010).

In the genus *Biomphalaria*, there are two different species complexes, whose members are practically indistinguishable by the morphology of their shells and by most characters of the genital system: (i) the *Biomphalaria straminea* complex, containing the species *B. straminea*, *Biomphalaria kuhniana* (Clessin, 1883) and *Biomphalaria intermedia* Paraense & Deslandes, 1962; and (ii) the *Biomphalaria tenagophila* complex, containing the species/subspecies *B. tenagophila tenagophila*, *Biomphalaria tenagophila guaibensis* Paraense, 1984 and *Biomphalaria occidentalis* Paraense, 1981 (Paraense, 1988; Caldeira et al., 1998; Spatz et al., 1999). Distinguishing between these taxa is one of the main challenges of research that aims to elucidate the phylogeny of the genus *Biomphalaria* using molecular techniques, such as the present work (Caldeira et al., 2000; Vidigal et al., 2004).

The study of the *Biomphalaria* genus has always been motivated by the epidemiological and ecological aspects of the relationship between these planorbids and *S. mansoni*. Despite this, the amount of research effort devoted to studies of the phylogeny and systematics of these groups has always been relatively small, even though elucidating the phylogenetic relationships of this group could change our understanding of its host-parasite relationships, which is fundamental for the development and planning of schistosomiasis control programs (Jarne et al., 2011).

Little is known about the evolution of the genus *Biomphalaria* due to the difficulty of placing fossils in the history of this species group, mainly because the morphometry of the shell is not used as an essential taxonomic characteristic, due to its simplicity and lack of relevant attributes regarding phylogeny (Cabrera et al., 2016). What can be said is that the evolution of mollusks of this genus is very recent, with the ancestral taxa within this genus beginning to split between the Pliocene and Pleistocene, around 1.8 million years ago, a fact that is consistent with the fossil record (Kaesler and Parodiz, 1970; Wanninger and Wollesen, 2019). Precisely for this reason, the characteristics used to distinguish between the species is quite limited; especially among closely-related species such as those of the *B. straminea* complex (Jarne et al., 2011). The morphological differences between *B. straminea* and *B. kuhniana* are very subtle and practically indistinguishable, with differences being limited to the number of muscular layers in the penis wall (three in *B. straminea* and two in *B. kuhniana*) (Paraense, 1988) and distal segment of the spermiduct very winding in *B. straminea* and straight or slighetly wavy in *B. kuhniana* (Paraense, 1988). Additionally, the molecular technique used for species differentiation within the genus (PCR/RFLP) generates a similar banding pattern between the two species (Caldeira et al., 1998), which creates several controversies in their differentiation.

The similarities between *B. tenagophila* and *B. occidentalis* are also numerous, again confusing human differentiation between, and identification of, the two species. For a long time, it was believed that the rates of infection by *S. mansoni* in the state of São Paulo were very low since infected mollusks were not found in the field. However, in 1981, the researcher Lobato Paraense described a new species, *B. occidentalis*, refractory to infection by the parasite, and that was previously confused with *B. tenagophila*, as they were almost indistinguishable by the shell morphology (Paraense, 1981). As a result of this description, in a large area in western Brazil, the distribution map of this last species has been undergoing constant change and readjustment (Scholte et al., 2012; Fernandez et al., 2014; Carvalho et al., 2020).

Traditionally, the DNA barcode technique consists of the amplification and sequencing of a fragment of the *cytochrome* c *oxidase I* (*coi*) gene, using pairs of universal primers – most often those proposed by Folmer et al. in 1994 – and subsequent comparison for specific identification with a universal database (BOLD) (Ratnasingham and Hebert, 2007). Therefore, the main objective of this work was to generate new DNA barcodes for the species of *Biomphalaria* found in Brazil, and to evaluate the use of the *coi* gene as a potential molecular marker for the identification of species of the genus *Biomphalaria*.

One of the main advantages of DNA barcoding is its ability to rapidly and accurately identify species, in the most variable life stages and genders, as well as identifying organisms by parts or pieces and discriminating individuals in a matrix with various mixed species (Casiraghi et al., 2010; Leray and Knowlton, 2015). It is also possible to identify species that are difficult to distinguish based on traditional morphological characteristics (Hebert et al., 2003; Casiraghi et al., 2010).

DNA barcoding is a useful tool for taxonomists, with good cost-effectiveness and the ability to allow non-experts to have access to quick and accurate identifications. This tool can be used in four ways (McManus et al., 2018): screening in collections, based on existing sequences in databases (James et al., 2018); identification of specimens where morphological identification is not possible (immature, partial, damaged or very small individuals), or even to resolve inconsistencies (in dimorphic species where identification is only possible in one sex) (Paéibo, 1990; Hebert et al., 2003; Casiraghi et al., 2010) (Jarne et al., 2010); as a powerful supplementary tool to other forms of identification and thus help in delimiting closely related species phylogenetically (Schindel and Miller, 2005; Sun et al., 2016); and (Corrêa, 1971) the possibility of discovering previously undescribed species (Hebert et al., 2004). In addition, DNA barcoding is a relatively low-cost and scalable technique, with the potential for high-throughput analysis of large numbers of samples. This makes it particularly useful for large-scale biodiversity surveys and monitoring programs (Hebert et al., 2003; Leray and Knowlton, 2015).

## 2 Methods

### 2.1 Construction of the database and illustrative maps

Nucleotide sequences of the partial region of the *coi* gene were obtained from a search of the public GenBank database (https://www.ncbi.nlm.nih.gov/genbank). Additional new sequences were obtained by us through DNA Sanger sequencing of mollusks deposited in the Medical Malacology Collection (Fiocruz-CMM) located at the Instituto René Rachou (IRR – Fiocruz/Minas). These specimens were selected according to the known geographic distribution of each species that occurs within Brazilian territory, prioritizing the type localities.

Data referring to the exact geographic coordinates of each collection point were added to each specimen used from Fiocruz-CMM and for sequences obtained from GenBank for which this information was available. For sequences in the database without any indication of the exact location of collection, the coordinates of the city in which they were collected were assigned.

From the coordinates and cartographic data of the IBGE (2021), a thematic map was constructed to illustrate the geographic distribution of the specimens in this study using the free software QGIS version 3.20 (QGIS, 2021).

### 2.2 Molecular studies

#### 2.2.1 - DNA extraction, amplification, and purification of the *coi* gene fragment

For specimens obtained from the Fiocruz-CMM collection, a cryopreserved fragment of the cephalopodal region was treated with proteinase K (0,1µg/µL) for at least 12 hours, precisely to degrade the proteins present in the mucus of the mollusk. Next, extraction is carried out with the commercial kit Wizard Genomic DNA Purification according to the manufacturer’s instructions

For the amplification of an ~600 bp region of the *coi* gene, the primers LCO1490 (5’-GGTCAACAAATCATAAAGATATTGG-3’) and HCO2198 (5’-TAAACTTCAGGGTGACCAAAAAATCA-3’) were used (Folmer et al., 1994). All PCR reactions were performed using the Platinum^®^ Taq DNA Polymerase kit (Invitrogen) following the manufacturer’s instructions and according to amplification conditions described by Folmer (Folmer et al., 1994).

DNA purification was performed using the *Wizard^®^ SV Gel and PCR Clean-Up System Kit* (Promega), according to the manufacturer’s instructions, using the PCR product directly in the purification columns. The purity and concentration of the purified PCR products were verified on a NanoDrop™ 2000 spectrophotometer (ThermoFisher Scientific).

#### 2.2.2 - Sequencing

The samples were sequenced by the Sanger method on an ABI 3730xL automatic sequencer (Applied Biosystems), using the same primers described above for amplification, at the Capillary Electrophoresis DNA Sequencing Platform of the IRR.

### 2.3 Data analysis

#### 2.3.1 Alignments

The reliability of the nucleotides obtained from sequencing was visually checked and verified using MEGA X software (Kumar et al., 2018) to obtain consensus sequences applying the MUSCLE algorithm (Edgar, 2004). Additionally, CodonCode Aligner^©^ v. 9.0.2 (CodonCode Corporation, 2019) was used to assign *Phred* quality values to each base. The *Phrap* program was then used to construct consensus sequences using the assigned quality values, with a minimum quality value of 20. The resulting consensus sequences were compared with those obtained by visual inspection to eliminate ambiguous bases. The consensus sequences were submitted to Nucleotide BLAST (BLASTn) (Altschul et al., 1990), and the BOLD species identification system (Ratnasingham and Hebert, 2007) (https://www.boldsystems.org/index.php/IDS_OpenIdEngine) to confirm the species identification and detect contamination with other organisms.

Sequences generated for *Biomphalaria* species in this study were combined with previously published sequences obtained from the GenBank database. MAFFT online version 7 (https://mafft.cbrc.jp/alignment/server/ (Katoh et al., 2019),) was employed to align all sequences. The resulting alignment was subsequently exported in FASTA format and visually inspected and edited in MEGA X. After trimming, the resulting sequences had sizes ranging from 517 to 655 bp.

#### 2.3.2 Phylogenetic reconstruction of the partial *coi* gene sequences

For the phylogenetic reconstruction, ModelFinder (Kalyaanamoorthy et al., 2017) implemented in *PhyloSuite* (Zhang et al., 2020) was used to find the evolutionary model that best fit the sequence alignment, according to the Corrected Akaike Information criterion (AICc).

Phylogenetic reconstruction was conducted using either Maximum Likelihood (ML) estimation (Felsenstein, 1981) or Bayesian Inference (BI) (Drummond et al., 2002). A *coi* sequence of the genus *Helisoma* sp. (Swainson, 1840), which also belongs to the Planorbidae family, was selected as an outgroup for tree rooting (GenBank accession number: KM612179.1).

ML trees were inferred using the IQ-TREE software v. 1.6.8 (Nguyen et al., 2015), with the evolutionary model K81u+R3+F for 5000 ultrafast *bootstraps* (Minh et al., 2013) and the Shimodaira-Hasegawa approximate likelihood ratio test (SH-aLRT) (Guindon et al., 2010) to determine the support values of the branches.

For BI the *MrBayes* software v. 3.2.6 (Ronquist et al., 2012) was used, with the evolutionary model HKY+I+G+F previously calculated by *ModelFinder* (Kalyaanamoorthy et al., 2017). Two independent parallel searches (Markov chains) were performed with 10^7^ generations (sampled every 1000 generations) and 25% of the initial data were discarded as burn-in.

The ML and BI trees were visualized and edited using the software *FigTree* v. 1.4.4 (Rambaut, 2018), with final editing performed using Inkscape v.1.1 (Inkscape Project, 2021).

#### 2.3.3 Verification of polymorphic sites and genetic divergence between populations

Genetic polymorphism indices were calculated for the partial region of the *coi* gene using the software *DnaSP* v. 6 (Rozas et al., 2017). The following indices of genetic diversity and their standard deviation were calculated: the number and diversity of mitochondrial haplotypes (h, Hd), nucleotide diversity (π) (Nei, 1987), both with and without the Jukes-Cantor correction (Jukes and Cantor, 1969; Nei, 1987; Lynch and Crease, 1990), the average number of differences between nucleotides between pairs of sequences (k) (Tajima, 1983; Nei, 1987). The number of singleton-type and parsimonious informative polymorphic sites were also estimated, in addition to the fixed divergence between populations (Hey, 1991) and nucleotide divergence between populations.

#### 2.3.4 Specimen identification and species delimitation

In order to verify the accuracy of the partial *coi* sequences for the identification of *Biomphalaria* specimens using DNA barcoding methodology, *BOLD Identification Criteria* (thresh-ID) and *Best Close Match* (BCM) analyses were carried out (Meier et al., 2006) using the *ape* v. 5.5 (Paradis and Schliep, 2019) and *spider* v. 1.5.0 (Brown et al., 2012) packages of the R software v. 4.0.5 (R Foundation for Statistical Computing, 2021).

In order to define the barcode gap, the Kimura two-parameter (K2P) model (Tamura et al., 2004) was used to calculate the intraspecific and interspecific distances (Meyer and Paulay, 2005) between the species using the *spider* v. 1.5.0 (Brown et al., 2012) package in the R software v. 4.0.5 (R Foundation for Statistical Computing, 2021).

Additionally, the sequences were clustered/grouped into Operational Taxonomic Units (OTUs), grouping organisms by their similarity, in this case in relation to a genetic marker (Blaxter et al., 2005), using the criteria of four algorithms; *Generalized Mixed Yule-Coalescent* (GMYC) (Reid and Carstens, 2012; Fujisawa and Barraclough, 2013), *Poisson Tree Processes* (bPTP) (Zhang et al., 2013), *Automatic Barcode Gap Discovery* (ABGD) (Puillandre et al., 2012) and *Assemble Species by Automatic Partitioning* (ASAP) (Puillandre et al., 2021).

## 3 Results

### 3.1 *coi* sequence database and specimen distribution map

Overall, the molecular data set analyzed consisted of 223 *Biomphalaria* nucleotide sequences, comprised of 83 previously published sequences obtained from GenBank (**Supplementary Table 1**) and 140 sequences newly-generated in this study (**Supplementary Table 2**). In addition, a single sequence was selected as an outgroup for phylogenetic analysis (*Helisoma* sp. - KM612179.1). The 11 species and one subspecies from Brazil are represented among the samples obtained, and all type localities are represented among the samples (except for the species *B. kuhniana*). The new sequences obtained in this study were deposited in GenBank (under Accession Numbers MZ778865 - MZ778963) and their IDs are listed in **Supplementary Table 2**.

**Figure 1:**
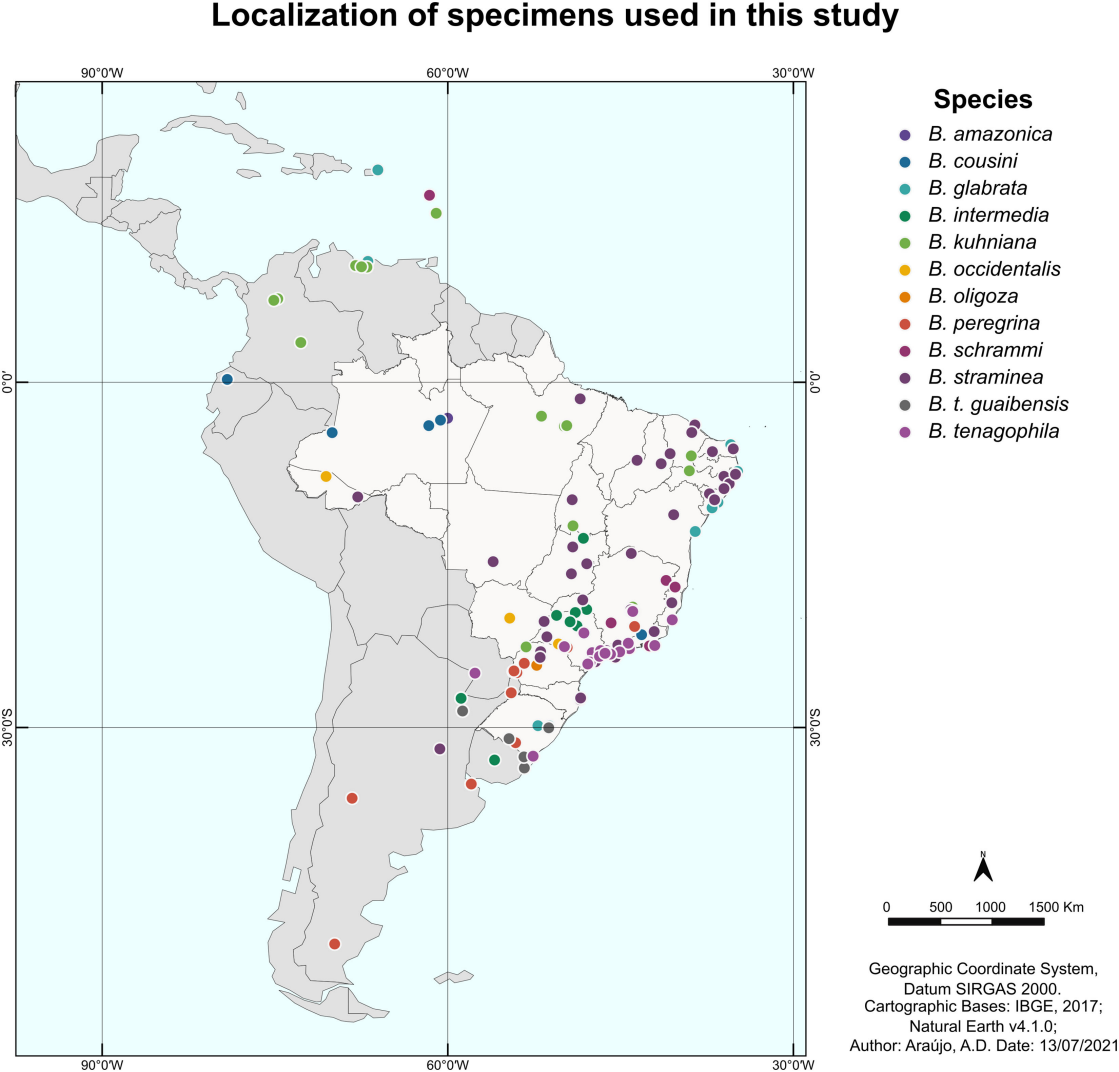
Map of Latin America showing the location of the specimens of the genus *Biomphalaria* used in this study. Each point represents the geographic location of a specimen, and the colors represent the different species used in the current study.

Based on the geographic location associated with each sequence, a map was constructed to illustrate the distribution in Central and South America of the specimens used in this study, not including those retrieved from GenBank databases located in Egypt and China (**Figure 1**).

### 3.2 Phylogenetic reconstruction of mollusks of the genus *Biomphalaria* using the partial region of the *coi* gene

**Figure 2:**
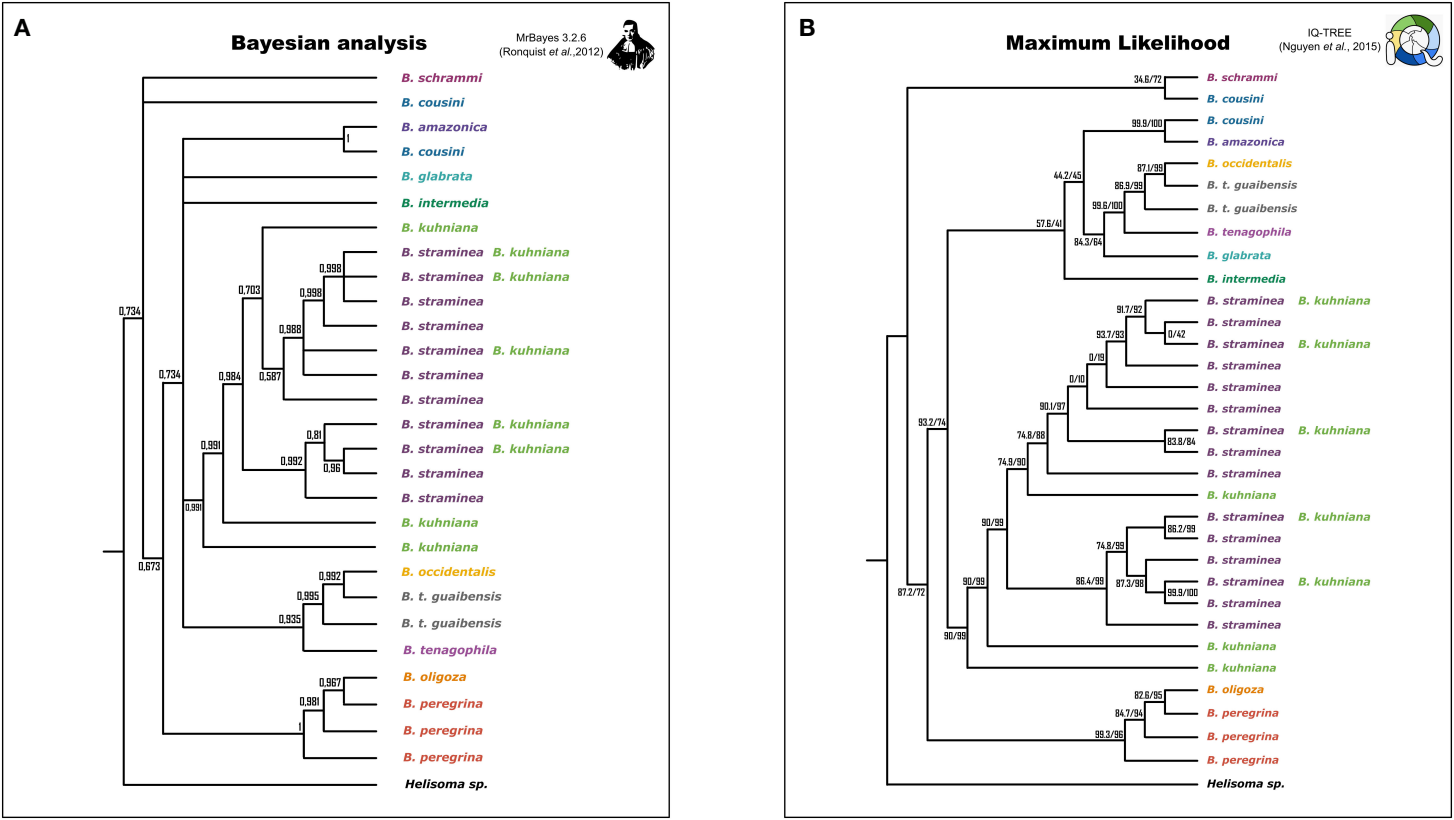
Simplified representation of the phylogenetic trees generated by Bayesian Inference (BI) and Maximum Likelihood (ML) criteria. Species are represented by different colors. Trees generated by MrBayes have posterior probability values represented on the branches **(A)**. Trees generated by IQ-TREE have two support values for comparison, ultrafast bootstraps (MINH et al., 2013) and the Shimodaira-Hasegawa test (SH-aLRT) (Guindon et al., 2010) **(B)**.

Phylogenetic inference produced well-supported clades that corresponded to the species studied, except for: (i) *B. cousini*, which formed two distantly-related lineages, one of which clustered with *B. amazonica*; (ii) *B. kuhniana* and *B. straminea*, whose sequences were polyphyletic, but together formed a single well-delimited monophyletic clade, within which the sequences of each of these two taxa were interspersed; (iii) The clades produced with sequences from *B. oligoza* and *B. peregrina* and *B. tenagophila guaibensis* were paraphyletic (**Figure 2**; **Supplementary Figure 1**). Since the topology of the trees are very similar, as well as the support values of the branches, we have provided only the representation of the tree from Bayesian Inference (Drummond et al., 2002) (**Supplementary Figure 1**).

### 3.3 Polymorphic sites and genetic divergence between *Biomphalaria* populations

Analysis of invariant and variable sites showed that most of the sites are invariant and most of the variable sites are parsimony-informative sites for most species (**Table 1**). The genetic polymorphism indices were also obtained for partial sequences of the *coi* gene from populations of *Biomphalaria* (**Table 2**).

**Table 1:** Polymorphic sites in the partial sequences of the *coi* gene from populations of *Biomphalaria*.

Species	Number of sequences analyzed	Invariant Sites (monomorphic)	Variable Sites (polymorphic)	Singleton type sites	Parsimonious information sites
All	223	312	193	25	168
*B. amazonica*	2	655	0	0	0
*B. cousini*	8	498	119	18	101
*B. glabrata*	22	502	44	12	32
*B. intermedia*	25	554	60	13	47
*B. kuhniana*	25	549	65	28	37
*B. occidentalis*	7	543	7	7	0
*B. oligoza*	3	586	2	2	0
*B. peregrina*	14	494	52	30	22
*B. schrammi*	8	541	60	36	24
*B. straminea*	70	481	56	15	41
*B. t. guaibensis*	8	504	13	0	13
*B. tenagophila*	31	527	22	6	16
*B. straminea* species complex	120	426	111	28	83
*B. tenagophila* species complex	46	469	48	7	41

Analysis of invariable and variable sites obtained using the DnaSP v. 6. The table describes the analyzed species, number of analyzed sequences, number of invariant (monomorphic) sites, number of variable (polymorphic) sites, and the numbers of singleton-type and parsimonious informative sites.

**Table 2:** Genetic polymorphism indices in partial sequences of the *COI* gene from populations of *Biomphalaria*.

Groups	Total number of mutations (η)	Total number of haplotypes (h)	Haplotype diversity (Hd)	Standard Deviation of Haplotype Diversity	Nucleotide diversity (π)	Nucleotide diversity (Jukes-Cantor), (π(JC))	Average number in nucleotide diversity (k)
All	273	119	0.987	0.0029	0.07235	0.07707	36.536
*B. amazonica*	0	1	0	0	0	0	0
*B. cousini*	126	6	0.929	0.084	0.09609	0.10693	59.286
*B. glabrata*	45	14	0.948	0.029	0.02331	0.02377	12.727
*B. intermedia*	66	18	0.977	0.016	0.02635	0.02698	16.180
*B. kuhniana*	69	20	0.98	0.017	0.02391	0.02438	14.683
*B. occidentalis*	7	4	0.714	0.181	0.00364	0.00366	2.000
*B. oligoza*	2	2	0.667	0.314	0.00227	0.00227	1.333
*B. peregrina*	53	11	0.956	0.045	0.0254	0.02604	13.868
*B. schrammi*	62	7	0.964	0.077	0.03417	0.03523	20.536
*B. straminea*	58	31	0.937	0.019	0.01718	0.01748	9.227
*B. t. guaibensis*	13	3	0.679	0.122	0.0143	0.01454	7.393
*B. tenagophila*	22	16	0.912	0.033	0.01119	0.01131	6.142
*B. straminea* species complex	124	58	0.969	0.008	0.03753	0.03896	20.156
*B. tenagophila* species complex	50	23	0.946	0.016	0.02606	0.02674	13.472

The table describes analyses of the total number of mutations (η), total number of haplotypes (h), haplotype diversity (Hd) and its standard deviation, nucleotide diversity (π), nucleotide diversity (Jukes-Cantor) (π(JC)), and the average number in nucleotide diversity (k) obtained using the software DnaSP v. 6.

The calculated divergence of DNA sequences between populations showed that: (i) there are no fixed differences between the species *B. amazonica* and *B. cousini*, (ii) fixed differences between the species within each of the different species complexes are smaller than those between these species and the other species which are not members of the species complex; and (iii) *B. schrammi* has the greatest number of differences when compared to most of the other species of the genus (**Supplementary Figure 2**). Also, in order to measure the distance between species, the average number of nucleotide divergences between populations (k) was calculated (**Supplementary Figure 3**). The greatest nucleotide divergences were observed for *B. cousini* and *B. schrammi*, with the divergence between the two species being the largest observed of the pairwise comparisons. The smallest nucleotide divergences from the pairwise comparisons were between the closest species already reported in the literature; that is, *B. kuhniana* and *B. straminea*, *B. t. guaibensis* and *B. occidentalis*, and *B. peregrina* and *B. oligoza*.

### 3.4 *Biomphalaria* species delimitation

**Figure 3:**
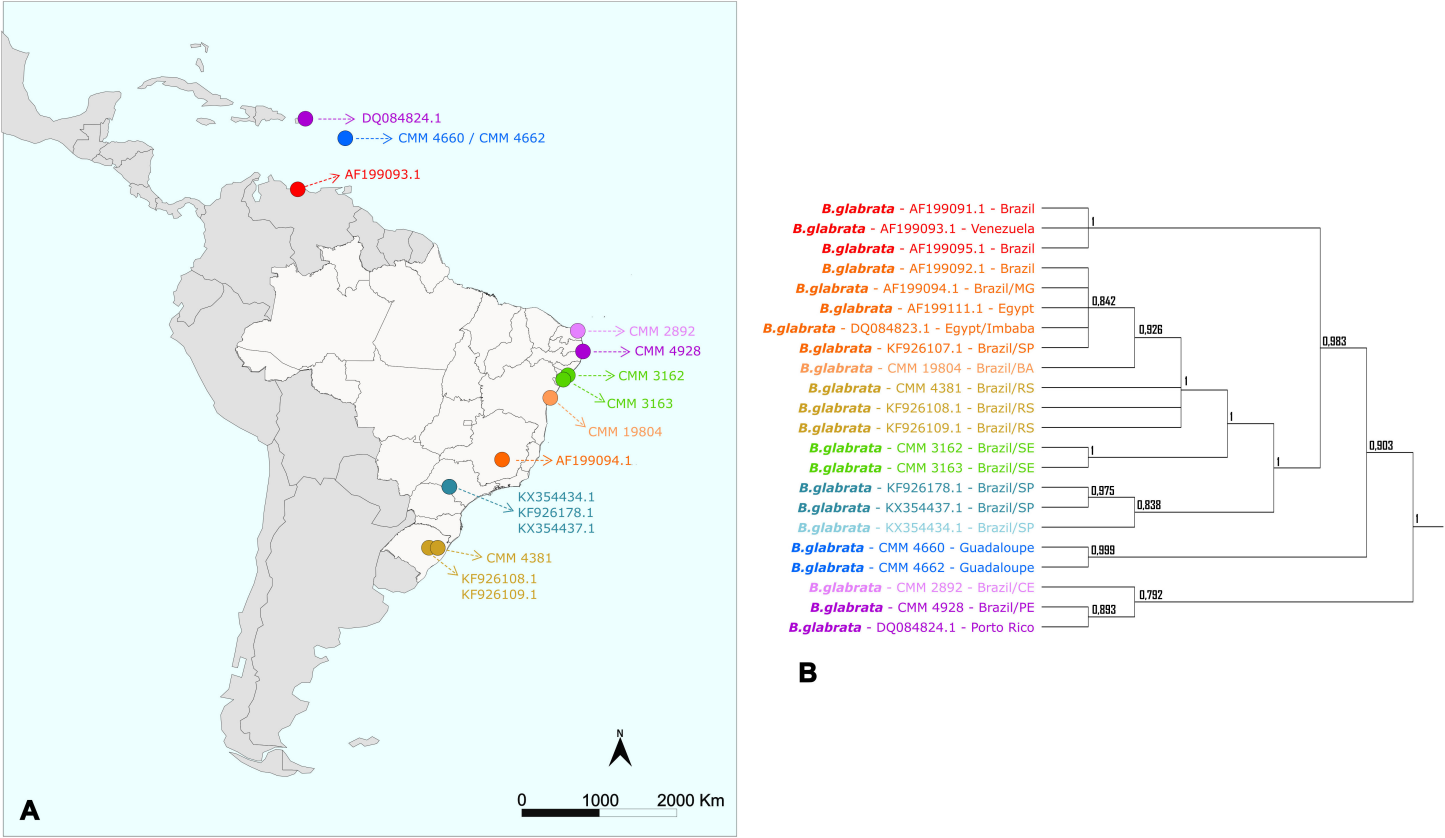
Number of sequences classified by the *Best Close Match* (BCM) and *BOLD Identification Criteria*. The bar graphs illustrate the absolute number of sequences identified using two different criteria: **(A)** *Best Close Match* (Meier et al., 2006) and **(B)** *BOLD Identification Criteria*, using the R software (version 4.0.5) and the package *spider* (version 1.5.0). The colors represent the four possible outcomes: correct (green), incorrect (red), ambiguous (yellow), and no identification (blue).

**Figure 4:**
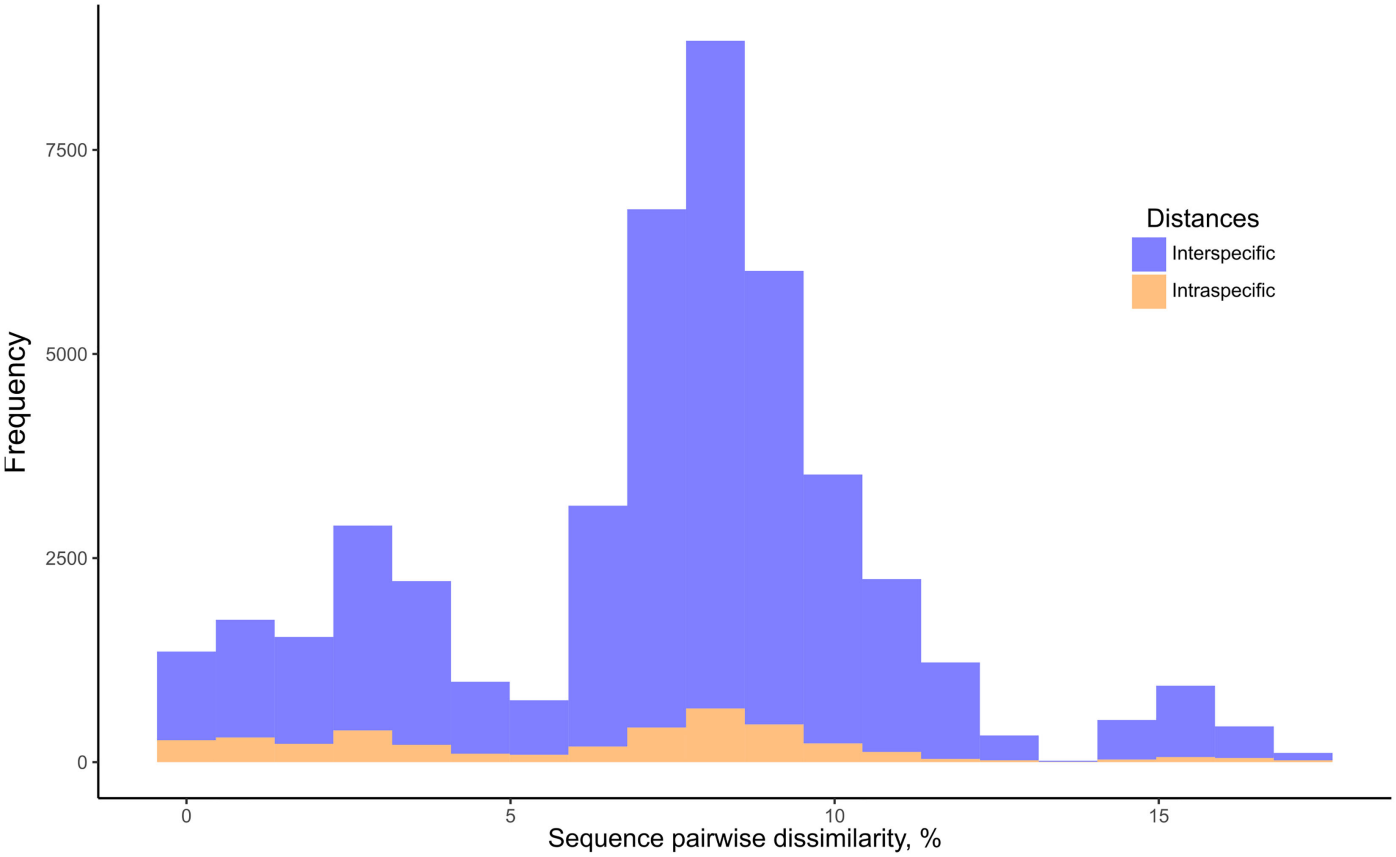
Frequency distribution of intraspecific and interspecific distances of *Biomphalaria* partial *coi* sequences. Histogram of intraspecific (orange) and interspecific (blue) distances based on the *Biomphalaria* partial *coi* sequences. Pairwise K2P distances were expressed as percentage dissimilarity (K2P * 100).

**Figure 5:**
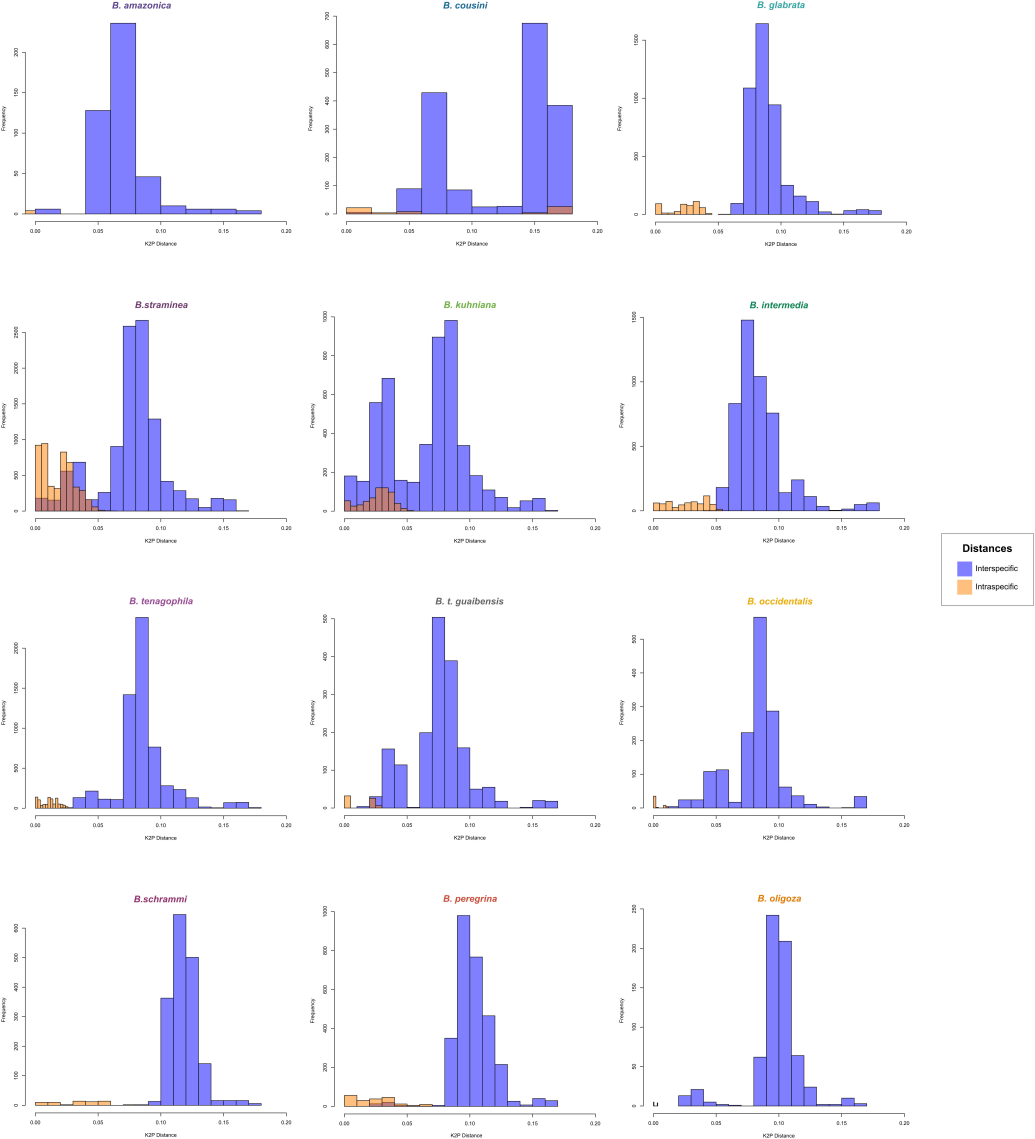
Frequency distribution of intraspecific and interspecific distances of *Biomphalaria* partial *coi* sequences according to species. Histogram of intraspecific (orange) and interspecific (blue) distances based on the *Biomphalaria coi* partial sequences. Pairwise distances were expressed as percentage dissimilarity for each species: *B. amazonica*, *B. cousini*, *B. glabrata*, *B. straminea*, *B. kuhniana*, *B. intermedia*, *B. tenagophila*, *B. t. guaibensis*, *B. occidentalis*, *B. schrammi*, *B. peregrina*, and *B. oligoza*.

*BOLD Identification Criteria* (thresh-ID) and *Best Close Match* (BCM) analyses showed similar results, with *B. tenagophila*, *B. glabrata*, *B. t. guaibensis*, *B. occidentalis*, and *B. oligoza* all showing 100% of the sequences correctly identified. In both analyses, *B. intermedia* had 24 (96%) correctly identified sequences, and one sequence without identification. *Biomphalaria peregrina*, had ten sequences correctly identified (71.4%), and four without identification. For *B. schrammi*, the two analyzes were able to correctly identify only two sequences (25%), and the other six sequences could not be identified. The biggest differences found between the two analyses were in the sequences of the two species *B. straminea* and *B. kuhniana* (**Figures 3A, B**). For *B. straminea*, using BCM analysis, 46 sequences were considered correct (65.7%), four were considered incorrect (5.7%), 19 were ambiguous (27.1%) and one sequence could not be identified. Using the BOLD analysis, 16 sequences were considered correct (22.9%), 53 were ambiguous (75.7%) and one could not be identified. For *B. kuhniana*, using the BCM analysis, 15 sequences were considered correct (60%), five incorrect (20%), two ambiguous (8%) and three without identification, while using the BOLD analysis, 15 sequences were considered correct (60%), three incorrect (12%), four ambiguous (16%) and three without identification.We calculated intra- and interspecific distances in order to define the barcode gap using the K2P model (**Figure 4**). The intraspecific distances calculated for all sequences presented a bimodal distribution, while the interspecific distances showed a trimodal distribution (**Figure 4**). The histograms of pairwise distances of each species (**Figure 5**) indicated the absence of a barcode gap for specific differentiation of *B. straminea B. kuhniana B. intermedia B. t. guaibensis B. occidentalis B. peregrina* and *B. oligoza*. Of the 223 analyzed sequences, it was possible to calculate the barcode gap for only 102, which corresponds to 45.7% of the analyzed sequences. Thus, 58.3% of the analyzed species have a barcode gap, which can be used to delimit them from one another using this methodology.

In the formation of Operational Taxonomic Units (OTU), different clusters/clades were generated for each algorithm used (**Table 3**). The clusters/clades generated by ABGD, ASAP, and sGMYC were the closest to the current delineation of the species of the *Biomphalaria* genus. Despite the large number of groups created by bPTP, and mGMYC, they were congruent with the geographic locality of the specimens. The ABGD algorithm divided *B. cousini* into three different groups, with one of them being shared with the *B. amazonica* sequences, as inferred by phylogenetic reconstruction. *Biomphalaria schrammi* and *B. peregrina* were also divided into two groups, the latter sharing a cluster with the *B. oligoza* sequences. The sequences of *B. kuhniana* and *B. straminea* were pooled, as well as *B. occidentalis*, *B. tenagophila*, and *B. t. guaibensis*. ASAP was the algorithm that generated the least number of clusters. However, *B. cousini* was partitioned into three different clusters by the ASAP method, with the same division observed for the ABGD algorithm. These latter two methods, therefore, generated groups, each comprised of multiple species, as follows. ABGD: (i) *B. amazonica* and *B. cousini*, (ii) *B. kuhniana* and *B. straminea*, (iii) *B. oligoza* and *B. peregrina*, and (iv) all three species of the *Biomphalaria tenagophila* complex (*B. occidentalis*, *B. tenagophila*, and *B. t. guaibensis*). ASAP: (i) *B. amazonica*, *B. cousini*, *B. intermedia*, *B. kuhniana*, and *B. straminea*, (ii) *B. oligoza* and *B. peregrina*, and (iii) all three species of the *Biomphalaria tenagophila* complex. Using the sGMYC algorithm, 14 groups were formed, as follows. *Biomphalaria cousini* was divided into two groups, with one of them shared with *B. schrammi* sequences, while the other group was shared with a single large group comprised of *B. amazonica*, *B. glabrata*, *B. intermedia*, *B. occidentalis*, *B. tenagophila* and *B. t. guaibensis*. *Biomphalaria kuhniana* was divided into six different groups, sharing four of them with *B. straminea*, which was itself divided into eight groups (**Supplementary Figure 1**).

**Table 3:** Number of Operational Taxonomic Units (OTUs) according to *Biomphalaria* species.

Species	ABGD	ASAP	sGMYC	mGMYC	bPTP
*B. amazonica*	1	1	1	1	2
*B. cousini*	3	3	2	5	6
*B. glabrata*	1	1	1	15	10
*B. intermedia*	1	1	1	15	16
*B. kuhniana*	1	1	6	20	20
*B. occidentalis*	1	1	1	5	1
*B. oligoza*	1	1	1	2	2
*B. peregrina*	2	1	1	10	10
*B. schrammi*	2	1	1	4	7
*B. straminea*	1	1	8	50	26
*B. tenagophila*	1	1	1	24	17
*B. t. guaibensis*	1	1	1	6	3
*TOTAL*	**11**	**7**	**14**	**153**	**118**

Table with the number of clusters generated by the species delimitation algorithms used in this study. The table shows the absolute number of clusters by species and overall for the entire data set of 223 sequences.

## 4 Discussion

The correct identification of mollusks that are the intermediate hosts of *Schistosoma* is a fundamental prerequisite for understanding the epidemiology of schistosomiasis and mobilizing efforts for disease surveillance and control (Rollinson et al., 2009). The Fiocruz-CMM combines both classical taxonomy through (i) comparison of morphological characters of the shell, as well as male and female reproductive organs (Lobato Paraense and Deslandes, 1958a; Lobato Paraense and Deslandes, 1958b; Paraense, 1975; Paraense, 1981; Paraense, 1984; Paraense, 1988; Paraense, 1990; Paraense et al., 1992; Estrada et al., 2006), and (ii) use of molecular tools, such as PCR-RFLP of the internal transcribed spacer (*ITS*) of the *ribosomal RNA* gene, to distinguish *Biomphalaria* species (Vidigal et al., 2000; Teodoro et al., 2010). Despite this, some inconsistencies between the results of the identifications have already been reported (Aguiar-Silva et al., 2014). This highlights the importance of combining methodologies in the taxonomy of mollusks of the genus *Biomphalaria* for species identification following the Iterative taxonomy process, in order to refine and define the boundaries between species using multiple different lines of evidence (Yeates et al., 2011).

This study confirmed that the species identification within the genus *Biomphalaria* is not a simple task, a fact that had already been mentioned by Doctor Lobato Paraense, in the 1960s (Paraense and Deslandes, 1959). The anatomy of the reproductive tract has been an effective tool in mollusk identification within the genus (Paraense and Deslandes, 1959). However, a resolution limit is reached for groups of closely related species (Paraense, 1981; Paraense, 1988), such as the *B. straminea* complex, *B. peregrina*/*B. oligoza*, and the *B. tenagophila* complex, as a consequence of intraspecific variation or incomplete variation, incomplete speciation, or the “gray zone of speciation” that exists between species due to recent evolution (De Queiroz, 2007; Jarne et al., 2011).

*Biomphalaria straminea* is a species that originated from the northern region of South America and has been expanding its habitat throughout Brazil and surrounding countries, mainly due to human activity and its high capacity to survive long periods of drought combined with its high fertility (Wang et al., 2013; Attwood et al., 2015). It is known that this species was recently introduced into both China (Meier Brook, 1975; Dudgeon and Yipp, 1983; Tang, 1983; Yipp, 1990; Yang et al., 2018), and the Caribbean (Paraense et al., 1981; Pointier et al., 1993) from strains originating from South America. The reconstructed phylogenies in this work reflect this introduction, with northeastern Brazilian specimens grouped with specimens from China, and specimens from the Caribbean grouped with specimens from northern Brazil, Venezuela, and Colombia.

In the reconstructed phylogeny, the *B. straminea* complex indicated a low resolution between *B. kuhniana* and *B. straminea*, which formed a polyphyletic clade. The same was observed between *B. cousini* and *B. amazonica*. This polyphyly may be the result of clades with recent evolution or imperfect taxonomy, in which not all the genus species diversity has been documented (Meyer and Paulay, 2005). Furthermore, due to the proximity between the species, there is a possibility that there is some level of hybridization between them (DeJong et al., 2001; Teodoro et al., 2011). Mitochondrial gene flow *via* hybridization has already been reported for several groups of animals, including planorbid mollusks (Mello-Silva et al., 1998), although it is not well understood. Hybridization is one of the sources of non-monophyly between clades of different groups of living beings, causing introgression (Anderson and Hubricht, 1938).

The clade representing the *B. glabrata* species is monophyletic and well supported (100%) in both the BI and ML analyses, with subdivision into at least five different groups. This fact can be explained by the Refuge Theory (Haffer, 1969), which suggests that climatic oscillations in the Pleistocene period may have been responsible for the habitat fragmentation and consequent geographic separation of *B. glabrata* populations, forming genetically-distinct clades (Dejong et al., 2003).

**Figure 6:**
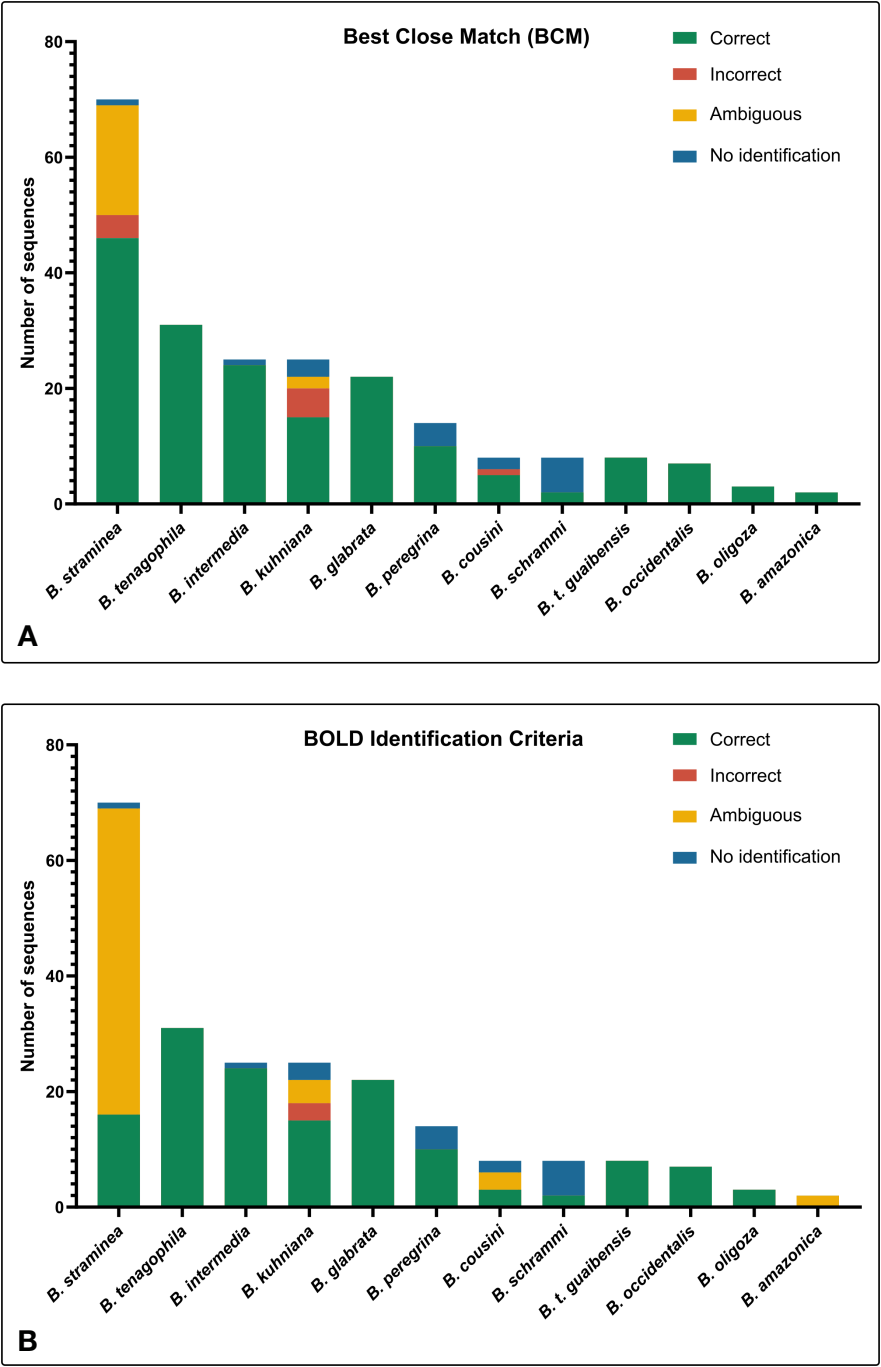
Distribution map and tree generated by Bayesian Inference of the *Biomphalaria glabrata* specimens used in this study. **(A)** Map with the geographical distribution of *Biomphalaria* specimens in Latin America. **(B)** Simplified phylogenetic tree obtained by Bayesian Inference (BI). The colors represent the large groups separated by the phylogeny of the species.

The phylogenetic trees inferred in this study corroborate what was indicated by previous studies that: (i) the Greater Antilles – Cuba, Hispaniola (Haiti and the Dominican Republic), Jamaica, and Puerto Rico – were colonized very early in *B. glabrata* evolution; (ii) specimens from southeastern Brazil were probably introduced into northeastern Brazil; and (iii) the Lesser Antilles and Venezuela appear to have been colonized by *B. glabrata* more recently, probably due to deforestation and human occupation (Mavárez et al., 2002; Dejong et al., 2003). Furthermore, as reported in 1998, our work also presents evidence of colonization of the South Region of Brazil with lineages from the North Region, probably related to recent human dispersal (**Figure 6**) (Carvalho O dos et al., 1998).

Of the 223 sequences used in this study, the barcode gap was successfully calculated for 102 (45.7%). However, of the 12 taxa studied, only seven (58.3%) can be differentiated from each other using DNA barcoding methodology. Among the invertebrate host species of *S. mansoni* in Brazil, only *B. straminea* could not be differentiated using this methodology, due to its genetic proximity to *B. kuhniana*. This result is promising regarding the use of DNA barcoding for taxonomy by the schistosomiasis epidemiology service in Brazil. In addition, due to the resolution observed using the *Best Close Match* (BCM) and *BOLD Identification Criteria* analyses, DNA barcoding methodology has a great potential to provide greater objectivity for mollusk identification, and can be used for the verification of identification performed by other methods (Ohlweiler et al., 2020). For species of the *B. tenagophila* complex, despite the proximity between the taxa, the DNA sequences were correctly identified by DNA barcoding methodology, with the analyses performed by *BOLD Identification Criteria* and *BCM*. Other authors also successfully separated the species of this complex, using DNA barcoding and phylogenetics, with high support values associated with each of the clades, and concluded that, for this species complex, molecular data may be more informative for species discrimination than morphology (Ohlweiler et al., 2020).

For some taxa, including *B. kuhniana* and *B. straminea*, the intraspecific divergence of the *coi* gene often surpassed the interspecific divergence, making coalescence methods for the formation of Operational Taxonomic Units (OTUs) inadequate for species delimitation. We observed the formation of many clusters within each species, some of them with only one sequence, others with sequences from different species within the same cluster, an artifact of the high variability observed for the *coi* gene. The use of nuclear markers with lower rates of evolution can be an alternative for the delimitation of species using these approaches.

The cluster analyses using species delimitation algorithms showed that the genetic divergence within populations was significant enough to generate substructuring within clades and differentiated clusters, which was also observed by other authors (Palasio et al., 2017). However, the genetic diversity observed among the species was not reflected in the morphological characters used in the classical taxonomy for distinguishing between species. Thus, it is evident that these morphological traits do not present enough characteristics to reflect the biodiversity of *Biomphalaria* in nature. The origin of such biodiversity inside the genus in South America may be a reflection of tropical forest fragmentation, which can cause speciation by allopatry result of the formation of distinct and isolated environments in recurrent episodes of vicariance (Colinvaux, 1987). This fragmentation of habitats has been substantially exacerbated by human intervention, particularly through the destruction of natural habitats through deforestation. (Barnosky et al., 2011).

The analysis of the variable sites revealed that *B. cousini* is the taxon with the highest number of polymorphic sites among the evaluated sequences. The number of polymorphic sites within the population indicates the intrapopulation genetic variability (Caldeira et al., 2001), showing that among all the analyzed species, *B. cousini* is the one that exhibits the greatest genetic diversity. Comparison of K2P genetic distances (Tamura et al., 2004) between the *B. cousini* sequences also showed values larger than 18%, values greater than the distance between this group and any other taxon in this study. One of the possible reasons for the diversity within this species may be the isolation of populations caused by the geographic barrier imposed by the Amazon rainforest, which also ends up preventing the dissemination of species from Venezuela, for example, *Biomphalaria prona* (Dejong et al., 2003; Jarne et al., 2011). Another relevant point for this discussion is that hybridization between *B. cousini* and *B. amazonica* has already been observed (Teodoro et al., 2011), which may have caused this high diversity among specimens of *B. cousini* and the proximity to *B. amazonica*, a fact already reported by Dr. Paraense in the description of the species, in 1966 (Paraense, 1966). These data may encourage discussion about the possibility of the existence of cryptic species within the currently recognized taxon *B. cousini*.

The systematics of mollusks is very nebulous and undergoes recurrent modifications (Wanninger and Wollesen, 2019). As observed for mussels of the genus *Pecten* (Canapa et al., 2000), this study showed that *B. straminea* and *B. kuhniana* are very similar, with small genetic distances, a fact that may raise the question of whether both may belong to the same species or are undergoing a recent speciation event.

## 5 Conclusions

Although the use of molecular data does not guarantee correct phylogenetic trees, and the study involving only one mitochondrial molecular marker is far from ideal and may not reliably reflect the evolution of the genus, this study demonstrated that the use of a region of the *coi* gene in the reconstruction of the phylogeny of the *Biomphalaria* genus can be an alternative to understanding the evolution and dispersion of the species. Despite the relationships established here, it must be considered that associating a single mitochondrial gene to the taxonomic history of a species is impossible, highlighting the need to expand studies with other molecular markers. The usefulness of the DNA barcoding methodology for mollusks of the genus *Biomphalaria* is well-suited to providing a better representation of this genus in public databases and the use of integrative methodologies for the correct identification of mollusks. This work contributed significantly to the number of partial sequences of the *coi* gene deposited in public databases, thus allowing a greater number of possible comparisons in future studies. Additionally, it was possible to obtain the correct delimitation of most of the *Biomphalaria* species whose occurrence is reported in Brazil using DNA barcoding and clustering/phylogenetic algorithms. Despite the high intraspecific diversity observed, two of the three species that act as intermediate hosts for *S. mansoni* in Brazil were successfully distinguished using the DNA barcoding technique based on the *coi* gene fragment. Further studies using both mitochondrial and nuclear markers are essential to elucidate the relationships between two of the most closely-related groups: *B. kuhniana* and *B. straminea*, and *B. cousini* and *B. amazonica*.

## Data availability statement

The datasets presented in this study can be found in online repositories. The names of the repository/repositories and accession number(s) can be found in the article/**Supplementary Material**.

## Author contributions

Conceptualization: RC, OC. Methodology & Investigation: AA, SG, RC. Formal analysis: AA, SG, RC. Resources: RC. Writing - original draft: AA. Writing – editing: AA, RC, SG, OC Writing – review: AA, RC, SG, OC. Visualisation: AA. Supervision: RC, SG. Project Administration: RC, SG. Funding acquisition: RC. All authors contributed to the article and approved the submitted version.
