# Supplementary material for: DNA barcoding as a valuable tool for delimiting mollusk species of the genus Biomphalaria Preston, 1910 (Gastropoda: Planorbidae)
Source: Front Cell Infect Microbiol. 2023 Apr 24;13:1167787. doi: 10.3389/fcimb.2023.1167787 (PMC10165093; doi:10.3389/fcimb.2023.1167787)
Supplement: Supplementary file 1 [file DataSheet_1.zip › Supplementary Material_DataSheets.pdf]

DNA barcoding as a valuable tool for delimitation of mollusk species of the genus *Biomphalaria* Preston, 1910 (Gastropoda: Planorbidae)

Supplementary Material

Supplementary Table 1. *coi* gene sequences obtained from GenBank. The table gives the GenBank accession number, species identification, geographic location, and reference of the *coi* gene sequences obtained from GenBank.

GenBank ID	Species	Country	State	Municipality	Authors
AF199091.1	*B. glabrata*	Brazil			Martin,S.K., Rollinson,D.
AF199092.1	*B. glabrata*	Brazil			Martin,S.K., Rollinson,D.
AF199093.1	*B. glabrata*	Venezuela			Martin,S.K., Rollinson,D
AF199094.1	*B. glabrata*	Brazil	MG	Belo Horizonte	Martin,S.K., Rollinson,D.
AF199095.1	*B. glabrata*	Brazil			Martin,S.K., Rollinson,D
AF199111.1	*B. glabrata*	Egypt			Martin,S.K., Rollinson,D
DQ084823.1	*B. glabrata*	Egypt	Imbaba		JØRGENSEN, *et al.*, 2007
DQ084824.1	*B. glabrata*	Puerto Rico			JØRGENSEN, *et al.*, 2007
**KF926107.1**	*B. glabrata*	Brazil	SE	Ilha das Flores	PALASIO *et al.*, 2017
KF926108.1	*B. glabrata*	Brazil	RS	Esteio	Tuan,R., Palasio,R.G.S., Zanna,R.D.
KF926109.1	*B. glabrata*	Brazil	RS	Esteio	Tuan,R., Palasio,R.G.S., Zanna,R.D.
KF926178.1	*B. glabrata*	Brazil	SP	Ourinhos	PALASIO *et al.*, 2017
KX354434.1	*B. glabrata*	Brazil	SP	Ourinhos	PALASIO *et al.*, 2017
KX354437.1	*B. glabrata*	Brazil	SP	Ourinhos	PALASIO *et al.*, 2017
KF926179.1	*B. intermedia*	Brazil	SP	São José do Rio Preto	PALASIO *et al.*, 2017
KJ137285.1	*B. kuhniana*	China	Guangdong	Shenzhen	ATTWOOD *et al.*,2015

KJ137287.1	*B. kuhniana*	China	Guangdong	Shenzhen	ATTWOOD *et al.*,2015
AF199090.1	*B. occidentalis*	Brazil			Martin,S.K., Rollinson,D
**KF926157.1**	*B. occidentalis*	Brazil	SP	Candido Mota	TUAN *et al.*, 2012
KF926163.1	*B. occidentalis*	Brazil	SP	Ourinhos	TUAN *et al.*, 2012
KF926174.1	*B. occidentalis*	Brazil	SP	Santa Isabel	PALASIO *et al.*, 2017
MH593413.1	*B. oligoza*	Brazil	SP	Biritiba Mirim	OHLWEILER *et al.*, 2020
MH593414.1	*B. oligoza*	Brazil	SP	Biritiba Mirim	OHLWEILER *et al.*, 2020
GU168593.1	*B. peregrina*	Argentina	Mendoza	Agua Escondida	Standley,C.J., Pointier,J.-P., Wisnivesky-Colli,C., Stothard,J.R.
JN621901.1	*B. peregrina*	Argentina	La Plata		COLLADO *et al.*, 2011
JN621902.1	*B. peregrina*	Argentina	La Plata		COLLADO *et al.*, 2011
JN621903.1	*B. peregrina*	Argentina	La Plata		COLLADO *et al.*, 2011
KF926176.1	*B. peregrina*	Brazil	SP	Presidente Prudente	Tuan,R., Ohlweiler,F.P., Palasio,R.G.S. and Guimaraes,M.C.A.
KX354439.1	*B. peregrina*	Brazil	RS	Bagé	PALASIO *et al.*, 2017
KX354441.1	*B. peregrina*	Brazil	SP	Ipaussu	PALASIO *et al.*, 2017
KX354443.1	*B. peregrina*	Brazil	SP	Ourinhos	PALASIO *et al.*, 2017
KY124272.1	*B. peregrina*	Argentina	Santa Cruz		Rumi,A., Vogler,R.E., Beltramino,A.A.
MK279703.1	*B. peregrina*	Argentina	Missiones	Salto El Mayno	VOGLER *et al.*, 2019
**MH593408.1**	*B. schrammi*	Brazil	SP	Pirapora do Bom Jesus	OHLWEILER *et al.*, 2020
AF199084.1	*B. straminea*	Brazil			Martin,S.K. and Rollinson,D.
AF199085.1	*B. straminea*	China	HongKong		Martin,S.K. and Rollinson,D.
KF926112.1	*B. straminea*	Brazil	SP	Araraquara	Tuan,R., Palasio,R.G.S, Zanna,R.D.
KF926117.1	*B. straminea*	Brazil	SP	Presidente Prudente	Tuan,R., Palasio,R.G.S, Zanna,R.D.
KF926118.1	*B. straminea*	Brazil	SP	Presidente Prudente	Tuan,R., Palasio,R.G.S, Zanna,R.D.

KF926184.1	*B. straminea*	Brazil	SP	Aparecida	PALASIO *et al.*, 2017
KF926185.1	*B. straminea*	Brazil	SP	Itariri	PALASIO *et al.*, 2017
KF926190.1	*B. straminea*	Brazil	SP	Santa Isabel	PALASIO *et al.*, 2017
KF926191.1	*B. straminea*	Brazil	SP	Ilha Bela	PALASIO *et al.*, 2017
KF926195.1	*B. straminea*	Brazil	SP	Santa Isabel	PALASIO *et al.*, 2017
KJ137289.1	*B. straminea*	China	Guandong	Shenzhen	ATTWOOD *et al.*,2015
KY697207.1	*B. straminea*	China	HongKong		Zeng,X.
KY697213.1	*B. straminea*	China	HongKong		Zeng,X.
KY697247.1	*B. straminea*	China	Guandong	Shenzhen	Zeng,X.
MF179807.1	*B. straminea*	China	Shenzhen		Zeng,X.
MF179827.1	*B. straminea*	China	Dongguan		Zeng,X.
MF179835.1	*B. straminea*	China	Shenzhen		Zeng,X.
MF179841.1	*B. straminea*	China	Shenzhen		Zeng,X.
MF179846.1	*B. straminea*	China	Shenzhen		Zeng,X.
MF179847.1	*B. straminea*	China	Dongguan		Zeng,X.
MF179848.1	*B. straminea*	China	Huiyang		Zeng,X.
MF491793.1	*B. straminea*	China	Guandong	Shenzhen	Habib,M.R., Guo,Y.-H., Lv,S., Zhou,X.-N
MF491794.1	*B. straminea*	China	Guandong	Shenzhen	Habib,M.R., Guo,Y.-H., Lv,S., Zhou,X.-N
MF491795.1	*B. straminea*	China	Guandong	Shenzhen	Habib,M.R., Guo,Y.-H., Lv,S., Zhou,X.-N
MH845638.1	*B. straminea*	China			Mao,Q.
NC036993.1	*B. straminea*	China			Wu,X. and Zhou,Y.
AF199089.1	*B. tenagophila*	Brazil			Martin,S.K. and Rollinson,D.
KF881853.1	*B. tenagophila*	Brazil	SP	Sorocaba	Tuan,R., Guimaraes,M.C.S., Palasio,R.G.S.
KF881854.1	*B. tenagophila*	Brazil	SP	Sorocaba	Tuan,R., Guimaraes,M.C.S., Palasio,R.G.S.

KF926133.1	*B. tenagophila*	Brazil	SP	Ourinhos	TUAN *et al.*, 2012
KF926148.1	*B. tenagophila*	Brazil	SP	Juquia	TUAN *et al.*, 2012
KF926149.1	*B. tenagophila*	Brazil	SP	Juquia	TUAN *et al.*, 2012
KF926152.1	*B. tenagophila*	Brazil	SP	Registro	TUAN *et al.*, 2012
KF926153.1	*B. tenagophila*	Brazil	SP	Registro	TUAN *et al.*, 2012
KF926154.1	*B. tenagophila*	Brazil	SP	Araraquara	TUAN *et al.*, 2012
KF926194.1	*B. tenagophila*	Brazil	SP	Ourinhos	PALASIO *et al.*, 2017
KF926202.1	*B. tenagophila*	Brazil	SP	Mogi das Cruzes	PALASIO *et al.*, 2017
KF926218.1	*B. tenagophila*	Brazil	SP	Caraguatatuba	PALASIO *et al.*, 2017
KF926220.1	*B. tenagophila*	Brazil	SP	Caraguatatuba	PALASIO *et al.*, 2017
KT225579.1	*B. tenagophila*	Brazil	SP	Juquia	PALASIO *et al.*, 2017
MF380474.1	*B. tenagophila*	Brazil	SP	Registro	Palasio,R.G.S. e Tuan,R.
MH593396.1	*B. tenagophila*	Brazil	SP	Biritiba Mirim	OHLWEILER *et al.*, 2020
MH593397.1	*B. tenagophila*	Brazil	SP	Biritiba Mirim	OHLWEILER *et al.*, 2020
MH593442.1	*B. tenagophila*	Brazil	SP	Suzano	OHLWEILER *et al.*, 2020
MH593451.1	*B. tenagophila*	Brazil	SP	Guarulhos	OHLWEILER *et al.*, 2020
MH593497.1	*B. tenagophila*	Brazil	SP	Franco da Rocha	OHLWEILER *et al.*, 2020
MH593504.1	*B. tenagophila*	Brazil	SP	Embu Guacu	OHLWEILER *et al.*, 2020
MH593506.1	*B. tenagophila*	Brazil	SP	Poa	OHLWEILER *et al.*, 2020
KF926156.1	*B. t. guaibensis*	Brazil	RS	Porto Alegre	TUAN *et al.*, 2012

Supplementary Table 2. Partial sequences of the *coi* gene obtained from Fiocruz-CMM samples selected for inclusion in this study. The table gives the GenBank accession number, CMM collection voucher, species identification, and geographic location of the Fiocruz-CMM samples selected for inclusion in this study.

GenBank ID	CMM Voucher	Species	Country	State	Municipality	Location
MZ778865	CMM 10462	*B. amazonica*	Brazil	AM	Manaus	Igarapé da Cachoeirinha, Centro. General Glicério Street 679, Cachoeirinha
MZ778866	CMM 12283	*B. amazonica*	Brazil	AM	Benjamin Constant	
MZ779002	CMM 10157	*B. cousini*	Brazil	MG	Santana do Deserto	Açude na Fazenda Santa Clara (Weir at Santa Clara Farm)
MZ778864	CMM 10425	*B. cousini*	Brazil	MG	Belo Horizonte	Moluscário Lobato Paraense (Snail Breeding Laboratory)
MZ779000	CMM 11223	*B. cousini*	Ecuador		Santo Domingo	Calle del Camal Municipal - El Elano, del Toachi.
MZ779001	CMM 11229	*B. cousini*	Ecuador		Santo Domingo	Calle del Camal Municipal - El Elano, del Toachi.
MZ778999	CMM 11583	*B. cousini*	Brazil	AM	Amori	Brejo, Sítio Alfredo - Vila Isabel (swamp at Alfredo Granje – Isabela Village)
MZ778868	CMM 3128	*B. cousini*	Brazil	AM	Benjamin Constant	
MZ778867	CMM 3956	*B. cousini*	Brazil	AM	Benjamin Constant	
MZ779003	CMM 5714	*B. cousini*	Brazil	AM	Manacapuru	Brejo - Estrada da correnteza (swamp – Correnteza Road)
MZ778979	CMM 19804	*B. glabrata*	Brazil	BA		Moluscário Lobato Paraense (Snail Breeding Laboratory)
MZ778983	CMM 2892	*B. glabrata*	Brazil	CE	Mirim	
MZ778981	CMM 3162	*B. glabrata*	Brazil	SE	Aracaju	
MZ778982	CMM 3163	*B. glabrata*	Brazil	SE	Aracaju	
MZ778980	CMM 4381	*B. glabrata*	Brazil	RS	Esteio	
MZ778985	CMM 4660	*B. glabrata*	Caribbean island	Guadaloupe		
MZ778986	CMM 4662	*B. glabrata*	Caribbean island	Guadaloupe		
MZ778984	CMM 4928	*B. glabrata*	Brazil	PE	Itamaraca	Sitio Bom Jesus (Bom Jesus Granje)

MZ778936	CMM 12469	*B. intermedia*	Brazil	MS	Três Lagoas	Lagoa Maior- Av. Aldair Rosa de Oliveira, em frente ao nº 1572, Centro/ Interlagoas (Lake)
MZ778939	CMM 12470	*B. intermedia*	Brazil	MS	Três Lagoas	Lagoa Maior- Av. Aldair Rosa de Oliveira, em frente ao nº 1572, Centro/ Interlagoas (Lake)
MZ778937	CMM 12471	*B. intermedia*	Brazil	MS	Três Lagoas	Lagoa Maior- Av. Aldair Rosa de Oliveira, em frente ao nº 1572, Centro/ Interlagoas (Lake)
MZ778941	CMM 12475	*B. intermedia*	Brazil	MS	Três Lagoas	Lagoa Maior- Av. Aldair Rosa de Oliveira, em frente ao nº 1175, Ipacarai (Lake)
MZ778940	CMM 12480	*B. intermedia*	Brazil	MS	Três Lagoas	Córrego da Onça- Vila Zuque (Onça Stream – Zuque Village)
MZ778959	CMM 13502	*B. intermedia*	Brazil	PR	Itaipulândia	Povoado Linha Lageado, Cedro (Linha Lageado Village)
MZ778951	CMM 2030	*B. intermedia*	Brazil	SP	Estrela D'Oeste	Trevo (city entrance)
MZ778956	CMM 2038	*B. intermedia*	Brazil	SP	Estrela D'Oeste	Trevo (city entrance)
MZ778938	CMM 2142	*B. intermedia*	Brazil	SP	Ariranha	Córrego Limoeiro (Limoeiro Stream)
MZ778947	CMM 2188	*B. intermedia*	Brazil	SP	Jales	Córrego Tanquinho I (Tanquinho I Stream)
MZ778958	CMM 2679	*B. intermedia*	Brazil	RS	Porto Alegre	
MZ778943	CMM 2871	*B. intermedia*	Brazil	MG	Patrocínio	Brejo Santa Terezinha (Santa Terezinha Swamp)
MZ778944	CMM 2874	*B. intermedia*	Brazil	MG	Patrocínio	Brejo Santa Terezinha (Santa Terezinha Swamp)
MZ778945	CMM 2875	*B. intermedia*	Brazil	MG	Patrocínio	Brejo Santa Terezinha (Santa Terezinha Swamp)
MZ778950	CMM 4167	*B. intermedia*	Argentina	Corrientes	Triangulo	
MZ778942	CMM 4168	*B. intermedia*	Argentina	Corrientes	Triangulo	
MZ778957	CMM 4180	*B. intermedia*	Uruguay			U4
MZ778952	CMM 4774	*B. intermedia*	Brazil	GO	Minaçu	Estrada para Palmeirópolis (city entrance)
MZ778954	CMM 4775	*B. intermedia*	Brazil	GO	Minaçu	Estrada para Palmeirópolis (city entrance)

MZ778953	CMM 4776	*B. intermedia*	Brazil	GO	Minaçu	Estrada para Palmeirópolis (city entrance)
MZ778955	CMM 4777	*B. intermedia*	Brazil	GO	Minaçu	Estrada para Palmeirópolis (city entrance)
MZ778948	CMM 7460	*B. intermedia*	Brazil	MG	Uberaba	Iturama - lote 1
MZ778949	CMM 7461	*B. intermedia*	Brazil	MG	Uberaba	Iturama - lote 1
MZ778946	CMM 9508	*B. intermedia*	Brazil	MG	Frutal	Córrego Frutal (Frutal Stream)
MZ778894	CMM 11316	*B. kuhniana*	Brazil	MG	Lagoa Santa	Parque Estadual do Sumidouro/Lagoa (State Park)
MZ778932	CMM 11558	*B. kuhniana*	Brazil	CE	Jati	
MZ778896	CMM 11665	*B. kuhniana*	Brazil	MG	Sabará	Lagoa da Reta (Lake)
MZ778897	CMM 11675	*B. kuhniana*	Brazil	CE	Icó	
MZ778870	CMM 13246	*B. kuhniana*	Brazil	PE	Sairé	Cedro – vala (ditch)
MZ778886	CMM 13426	*B. kuhniana*	Brazil	PR	Santa Izabel do Ivaí	Fazenda Volta Grande (Volta Grande Farm)
MZ778883	CMM 18837	*B. kuhniana*	Brazil	MG	Uberlândia	Córrego Bons Olhos (Bons Olhos stream)
MZ778895	CMM 19687	*B. kuhniana*	Brazil	TO	Alvorada	Lagoa Sazonal (Sazonal lake)
MZ778920	CMM 2490	*B. kuhniana*	Venezuela	Aragua	Villa De Cura	Pao river
MZ778911	CMM 3260	*B. kuhniana*	Brazil	PA	Tucuruí	Lagoa localizada na Rua Alto Alegre *B.* Cohab (Lake at Alto Alegre Street)
MZ778912	CMM 3318	*B. kuhniana*	Brazil	PA	Tucuruí	Santo Antonio Street
MZ778921	CMM 3810	*B. kuhniana*	Venezuela	Carabobo	Valência	Laguna del Parque Recreacional (Lake)
MZ778928	CMM 3869	*B. kuhniana*	Venezuela	Aragua	San Casimiro	La Barquera, el Loro Rio
MZ778931	CMM 3942	*B. kuhniana*	Venezuela	Aragua	Villa De Cura	Balzanito quebrado, rio Guarico
MZ778927	CMM 4785	*B. kuhniana*	Colombia	Antioquia	Segovia	
MZ778923	CMM 5555	*B. kuhniana*	Colombia	Antioquia		Llano Grande
MZ778925	CMM 5557	*B. kuhniana*	Colombia	Antioquia		Llano Grande

MZ778924	CMM 5595	*B. kuhniana*	Colombia	Meta		
MZ778926	CMM 5746	*B. kuhniana*	Brazil	AC	Rio Branco	Calafate brejo (Calafate swamp)
MZ778930	CMM 5747	*B. kuhniana*	Brazil	AC	Rio Branco	Calafate brejo (Calafate swamp)
MZ778929	CMM 5794	*B. kuhniana*	Caribbean island	Guadaloupe		Romain Pond
MZ778922	CMM 5832	*B. kuhniana*	Caribbean island	Martinique		Fond Bellemare River
MZ778913	CMM 9633	*B. kuhniana*	Brazil	PA	Carajás	
MZ778960	CMM 11450	*B. occidentalis*	Brazil	AC	Tarauacá	Igarapé Preto Road, Km 3 , Fausto Soares Farm
MZ778961	CMM 12435	*B. occidentalis*	Brazil	MS	Campo Grande	Lagoa do Amor. Av. Senador Filinto Muller, bairro Ipiranga (Lagoon at Senador Filinto Muller Avenue)
MZ778962	CMM 12483	*B. occidentalis*	Brazil	MS	Três Lagoas	Valas de Dreno do Cinturão Verde - Sítio Nossa Senhora Aparecida (drain ditches at Nossa Senhora Aparecida Granje)
MZ778987	CMM 13338	*B. oligoza*	Brazil	PR	Roncador	Novo Alto São João
MZ778991	CMM 10016	*B. peregrina*	Brazil	MG	Barbacena	Village (Pinheiro Grosso)
MZ778989	CMM 13466	*B. peregrina*	Brazil	PR	Matelândia	Village (Alto Dourado)
MZ778990	CMM 13487	*B. peregrina*	Brazil	PR	Missal	Comunidade Esquina Gaúcha (Village)
MZ778988	CMM 14510	*B. peregrina*	Brazil	PR	Iracema do Oeste	Povoado Estrada Alvorada (Vllage)
MZ778992	CMM 15475	*B. schrammi*	Brazil	MG	Crisolita	Córrego afluente do pratinha (Pratinha stream)
MZ778993	CMM 15528	*B. schrammi*	Brazil	MG	Serra dos Aimorés	Córrego Estivinha Fazenda Sol Nascente (Estivinha stream – Sol Nascente Farm)
MZ778994	CMM 3379	*B. schrammi*	Brazil	MG	Ilicinea	Lagoa e Brejo (Lagoon and Swamp)
MZ778998	CMM 4884	*B. schrammi*	Brazil	RJ	Saquarema	
MZ778996	CMM 5782	*B. schrammi*	Caribbean island	Guadaloupe		Bazin Pond (lagoon)
MZ778995	CMM 5789	*B. schrammi*	Caribbean island	Guadaloupe		Bazin Pond (lagoon)
MZ778997	CMM 7130	*B. schrammi*	Brazil	SE	Propriá	
MZ778888	CMM 10647	*B. straminea*	Brazil	SP	Caraguatatuba	Vala do Bairro Jetuba (Ditch at Jetuba Neighborhood)

MZ778933	CMM 10694	*B. straminea*	Brazil	PI	Guadalupe	Vila Parnaíba (Parnaíba Village)
MZ778889	CMM 10835	*B. straminea*	Brazil	PR	Maringá	Lagoon at Esperança Neighborhood
MZ778908	CMM 12344	*B. straminea*	Brazil	RN	Jucurutu	Jucurutu tank
MZ778902	CMM 12479	*B. straminea*	Brazil	MS	Três Lagoas	Spring Conturão Verde in the Jupiá neighborhood
MZ778903	CMM 12487	*B. straminea*	Brazil	TO	Palmas	Graciosa Beach - Hydroelectric Power Plant Lake
MZ778904	CMM 12491	*B. straminea*	Brazil	TO	Palmas	Graciosa Beach - Hydroelectric Power Plant Lake
MZ778901	CMM 12493	*B. straminea*	Brazil	TO	Palmas	Graciosa Beach - Hydroelectric Power Plant Lake
MZ778906	CMM 12494	*B. straminea*	Brazil	TO	Palmas	Ribeirão do Taquarucú Lagoon - Beach behind the airport
MZ778905	CMM 13333	*B. straminea*	Brazil	PR	Fenix	Santa Terezinha Farm
MZ778869	CMM 13691	*B. straminea*	Brazil	PE	Caruarú	Murici II - Ditch
MZ778875	CMM 13695	*B. straminea*	Brazil	PE	Caruarú	Alto do Moura - Ipojuca River
MZ778881	CMM 14593	*B. straminea*	Brazil	MG	Ribeirão das Neves	Alterosa - Lagoon
MZ778899	CMM 15391	*B. straminea*	Brazil	CE	Parambu	Location Cachimbo
MZ778871	CMM 15592	*B. straminea*	Brazil	RJ	Itaocara	
MZ778877	CMM 15887	*B. straminea*	Brazil	PE	Santa Terezinha	Tigre Village - lakes and dams
MZ778915	CMM 15975	*B. straminea*	Brazil	ES	São Domingos do Norte	São Domingos stream, downtown, close to Dario Faquete
MZ778876	CMM 16183	*B. straminea*	Brazil	AL	Branquinha	Calimba - Santa Tereza Farm- water tank with spout
MZ778872	CMM 16863	*B. straminea*	Brazil	AL	Jacaré dos Homens	
MZ778879	CMM 18451	*B. straminea*	Brazil	MG	Belo Horizonte	Santa Lucia Dam - Lagoon
MZ778900	CMM 18771	*B. straminea*	Brazil	MT	Cuiabá	Tia Nair Park Fountain - Eurico Preza Avenue - Jardim Itália neighborhood
MZ778882	CMM 18839	*B. straminea*	Brazil	MG	Uberlândia	Bons Olhos Stream

MZ778916	CMM 18950	*B. straminea*	Brazil	ES	Baixo Guandu	Mascarenhas 1
MZ778918	CMM 2613	*B. straminea*	Brazil	BA	Varzea do Poço	
MZ778919	CMM 2614	*B. straminea*	Brazil	BA	Varzea do Poço	
MZ778890	CMM 2634	*B. straminea*	Brazil	PA	Belém	Lobato
MZ778887	CMM 3361	*B. straminea*	Brazil	DF	Brasília	Fumal Capitation Station - CAESB Brasília Water and Sewerage Company
MZ778914	CMM 3364	*B. straminea*	Brazil	DF	Brasília	Fumal Capitation Station - CAESB Brasília Water and Sewerage Company
MZ778893	CMM 3669	*B. straminea*	Brazil	SC	Florianópolis	Alto Ribeirão - Marcelo Filomeno Highway - Stream under the bridge
MZ778891	CMM 3735	*B. straminea*	Brazil	SC	Florianópolis	Entrance of Canasvieiras Cardoso Vieira – 11 Street
MZ778880	CMM 4305	*B. straminea*	Argentina		Espinillar	
MZ778907	CMM 4763	*B. straminea*	Brazil	GO	Campinorte	Planeta Água Farm
MZ778935	CMM 4806	*B. straminea*	Brazil	PI	Picos	
MZ778910	CMM 4934	*B. straminea*	Brazil	PE	São Lourenço da Mata	Engenho Camorim
MZ778898	CMM 5272	*B. straminea*	Brazil	CE	Fortaleza	Aquario e Companhia
MZ778909	CMM 5280	*B. straminea*	Brazil	CE	Aracoiaba	Dam of the Aracoiaba River
MZ778934	CMM 5292	*B. straminea*	Brazil	CE	Aracoiaba	Dam of the Aracoiaba River
MZ778878	CMM 5729	*B. straminea*	Brazil	AC	Rio Branco	São Francisco River - Santa Inês street (São Francisco neighborhood)
MZ778885	CMM 5768	*B. straminea*	Brazil	AC	Rio Branco	State Fish Farming Station
MZ778884	CMM 5993	*B. straminea*	Brazil	GO	Goiânia	Senador Gomes - Water tank, Retiro Farm
MZ778874	CMM 6535	*B. straminea*	Brazil	RN	Natal	Drain Ditches
MZ778873	CMM 7115	*B. straminea*	Brazil	SE	Propriá	Pau das Marrecas
MZ778892	CMM 7118	*B. straminea*	Brazil	SE	Propriá	Pau das Marrecas

MZ778917	CMM 9094	*B. straminea*	Brazil	MG	São João das Missões	Itacarambi stream (near the bridge)
MZ778970	CMM 10473	*B. tenagophila*	Brazil	MG	Contagem	Jardim Público - José Américo Cansado Bahia street - near number 1045 – Barraginha Village
MZ778976	CMM 10902	*B. tenagophila*	Brazil	RJ	Angra dos Reis	Abraão Village - Ilha Grande
MZ778977	CMM 11370	*B. tenagophila*	Brazil	SP	Bananal	Elivelton Martins Granje
MZ778978	CMM 11372	*B. tenagophila*	Brazil	SP	Bananal	Elivelton Martins Granje
MZ778972	CMM 1146	*B. tenagophila*	Paraguai		Asunción	
MZ778974	CMM 16968	*B. tenagophila*	Brazil	MG	Belo Horizonte	Moluscário Lobato Paraense (Snail Breeding Laboratory)
MZ778971	CMM 7080	*B. tenagophila*	Brazil	ES	Guarapari	
MZ778975	CMM 8043	*B. tenagophila*	Brazil	RJ	Cabo Frio	
MZ778973	CMM 8048	*B. tenagophila*	Brazil	RS	Taim	
MZ778968	CMM 4282	*B. t. guaibensis*	Argentina	Corrientes	San Roque	
MZ778969	CMM 4298	*B. t. guaibensis*	Argentina	Corrientes	San Roque	
MZ778967	CMM 4327	*B. t. guaibensis*	Argentina	Corrientes	San Roque	
MZ778964	CMM 6549	*B. t. guaibensis*	Brazil	RS	Santa Vitória Palmar	Curral alto
MZ778965	CMM 8936	*B. t. guaibensis*	Brazil	RS	Dom Pedrito	Passinho do Amor
MZ778966	CMM 8966	*B. t. guaibensis*	Brazil	RS	Jaguarão	Bretanhas - Irrigation channel
MZ778963	CMM 9587	*B. t. guaibensis*	Brazil	RS	Taim	BR 471 Road, near Km 490 - Same side as headquarters

Supplementary Figure 1. Phylogenetic tree generated by Bayesian Inference using MrBayes version 3.2.6 (RONQUIST *et al.*, 2012). Species are represented by different colors. Values close to the nodes represent the support values of the Bayesian Inference. The clusters formed by the ABGD, ASAP and sGMYC algorithms are represented in different colors.

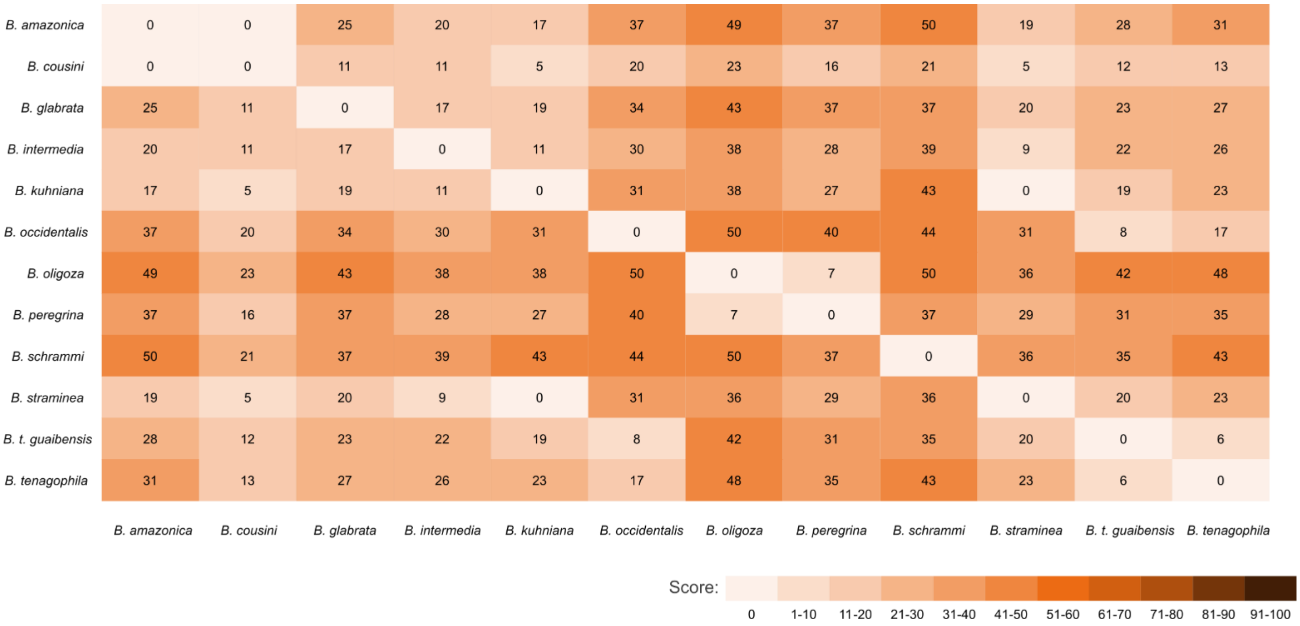

Supplementary Figure 2. Relationships between *Biomphalaria* species according to fixed differences observed in the partial sequences of the *coi* gene. Heatmap that illustrates the fixed differences between the Brazilian species of the genus *Biomphalaria*, from the analysis of DNA divergence between sample sequences of each population using DnaSP v. 6.

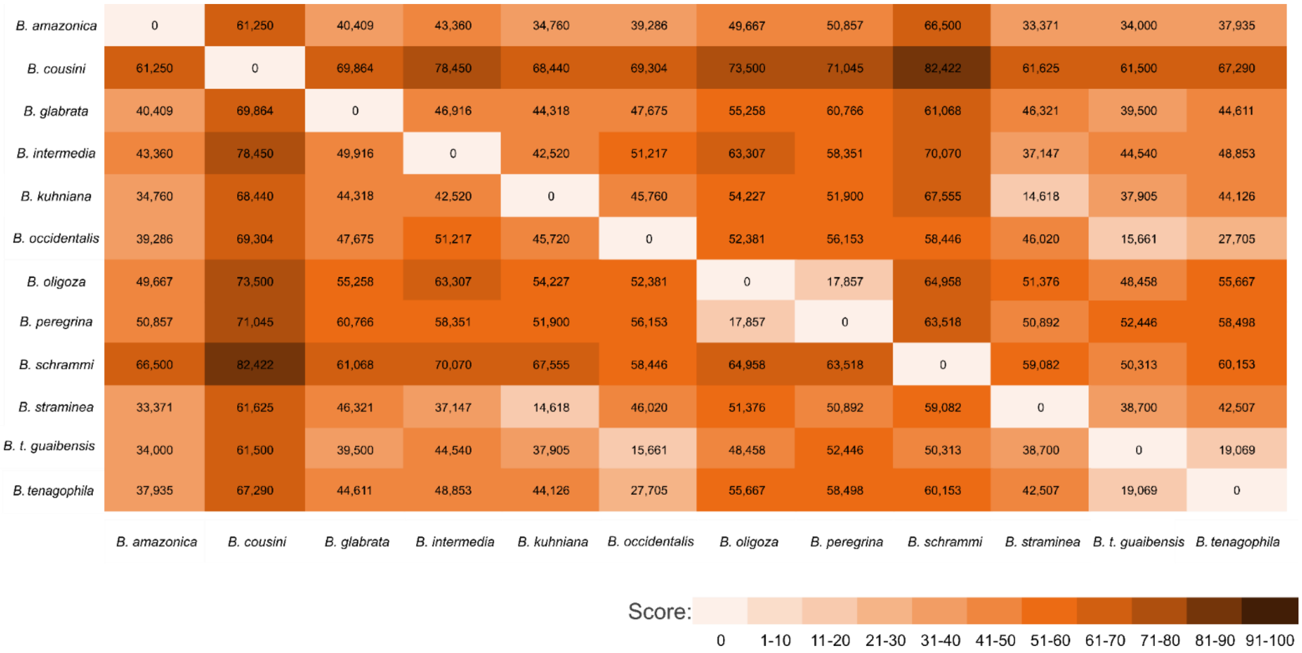

Supplementary Figure 3. Relationships between *Biomphalaria* species according to the average number of nucleotide differences between populations (k) observed in the partial sequences of the *coi* gene. Heatmap that shows the average number of nucleotide differences between populations (k) among the Brazilian species of the genus *Biomphalaria*, from the analysis of DNA divergence between sequence samples of each population using DnaSP v. 6.
